# Analysis of Characteristics and Driving Mechanisms of Non-Grain Production of Cropland in Mountainous Areas at the Plot Scale—A Case Study of Lechang City

**DOI:** 10.3390/foods13101459

**Published:** 2024-05-08

**Authors:** Zhaojun Wu, Shengfa Li, Dafang Wu, Jun Song, Tong Lin, Ziya Gao

**Affiliations:** 1 Guangzhou Institute of Geography, Guangdong Academy of Sciences, Guangzhou 510070, China; 2112201066@e.gzhu.edu.cn (Z.W.); sj20010226@gmail.com (J.S.); 2 School of Geography and Remote Sensing, Guangzhou University, Guangzhou 510006, China; wudafang@gzhu.edu.cn (D.W.); 2112301076@e.gzhu.edu.cn (Z.G.); 3 School of Government Management, Beijing Normal University, Beijing 100875, China; 202331260010@mail.bnu.edu.cn

**Keywords:** non-grain production on cropland, food security, clustering characteristics, distribution characteristics, driving mechanisms, measurement index, typological characteristics

## Abstract

With the continuous advancement of urbanization and industrialization, non-grain production on cropland (NGPCL) is gradually becoming more widespread. This phenomenon will compress the space for grain production and trigger a global food crisis. How to scientifically understand and effectively control NGPCL has become a scientific issue. This study, conducted at the plot scale, establishes a measurement index for NGPCL and further explores the characteristics and driving mechanisms of NGPCL in mountainous areas. The results indicate the following: (1) Compared to plots for food-growing, plots for non-grain production tend to be more dispersed, with terraces showing the most significant dispersion, while plains exhibit the highest degree of aggregation. (2) In terms of irrigation conditions, irrigated land and dry land are more likely to undergo NGPCL. In terms of topography, slope croplands have the highest probability of being used for NGPCL. (3) Regions with steeper slopes, higher elevations, greater differences in altitude from the settlement, farther distances from settlements and roads, yet closer proximity to forests, are more likely to engage in NGPCL. (4) Different plot characteristics have varying impacts on NGPCL. Plot characteristics primarily affect the costs and returns of grain production, driving farmers to change their production patterns and triggering NGPCL.

## 1. Introduction

Food security is essential for ensuring there is a basic guarantee to meet human food demand and is also an important foundation for global sustainable development [1]. Under the coordination of the United Nations, countries around the world have jointly taken the most effective action in history to eradicate poverty and hunger, achieving significant results [2,3]. However, in reality, food issues remain a focus of international attention. Although the incidence of food shortages in developing countries has decreased from 23.3% in 1990 to 8.3% in 2015, there are still 265 million people suffering from severe hunger globally, indicating that the issue of food insecurity remains severe [4,5]. In recent years, factors such as extreme weather and the Ukraine crisis have further worsened the food security situation, and the continuous growth of the global population has brought ever greater pressure on the provision of food for all humanity [6,7].

On a global scale, with the continuous advancement of urbanization and industrialization, there have been drastic changes in the utilization pattern of cropland, which has had a significant impact on food security [8]. On the one hand, to meet the demand for construction land due to urban development, a large amount of cropland has been converted into construction land [9]. On the other hand, due to further increased profitability of other agricultural production activities, as well as the continuous increase in input costs and labor costs for grain production, an increasing number of farmers are withdrawing from grain production [10,11], leading to the emergence of non-grain production on cropland (NGPCL). NGPCL primarily refers to all activities conducted on cropland that do not involve the production of grain. This includes planting cash crops, as well as engaging in agricultural activities such as aquaculture, animal husbandry, and fallowing of cropland [12,13,14]. Although NGPCL does not cause explicit loss of cropland quantity, it implicitly reduces the grain output capacity of cropland, posing a serious threat to regional food security [15]. Influenced by factors such as urbanization and dietary structure upgrades among residents, NGPCL in China is becoming increasingly common [16]. As China contributes approximately 50% of the grain output in Asia, and its share of grain imports in global grain trade is close to 30% [10,17], China serves as a crucial node in world grain trade [18,19]. Therefore, the phenomenon of NGPCL in China has attracted widespread attention from the international community.

In 2019, the area of cropland used for non-grain production in China accounted for 29.06% of the total cropland area [20], and by 2021 this figure had increased to 33.2% [21]. NGPCL is more prevalent in the southern and eastern regions of China. In the southwest region, due to significant loss from the agricultural labor force, there is frequent fallowing of cropland in mountainous areas, and a large amount of cropland is also used for planting economic forests. According to statistics, 42.87% of cropland in Chongqing Municipality is not used for planting grain [7,12]. In the southeast region, due to the rising cost of labor in the labor market and the increasing demand for cash crops, the main crops have shifted from grains to vegetables and other oil crops. More than 40% of cropland resources have been used for non-grain production [22,23]. From 2015 to 2021, China’s annual average sown area of grain crops decreased by 0.4%, with the planting area of rice and wheat decreasing by 8629 km^2^ and 9985 km^2^, respectively [24]. Although the improvement in grain production efficiency in China has compensated for the impact of the decrease in grain production area on grain output, breakthroughs in production technology are inevitably facing bottlenecks. The compression of grain production space caused by NGPCL will pose a serious threat to domestic grain output and affect global food supply [25].

Although importing food can alleviate the pressure on regional food production, from a global perspective, the total amount of cropland resources is limited. Extensive NGPCL on a large scale is bound to increase the pressure on the global food supply. Considering the serious impact of NGPCL, there has been a gradual increase in research on this topic. Currently, there is no clear academic concept for NGPCL. Foreign scholars typically approach a related analysis from the perspective of agricultural sustainable development, discussing the theoretical logic and specific practices of diversified planting [26,27,28]. Chinese scholars were among the first to regard NGPCL as an independent utilization method for cropland, and they have conducted discussions around the spatial characteristics and influencing factors of NGPCL. Scholars commonly measure the level of NGPCL within research units based on indicators such as the proportion of cropland area planted with cash crops or the proportion of cropland area planted with cash crops amid transferred cropland [22,29]. Moreover, scholars often use administrative regions as research units and employ geographical analysis models, such as spatial autocorrelation and standard deviation ellipses, to explore the spatial patterns of NGPCL [30,31,32]. Research indicates that hilly and mountainous areas are prominent regions for NGPCL, and cropland in economically developed areas is more likely to be used for non-grain production [33,34]. Cropland near urban areas is generally not cultivated for grain crops [14]. In terms of the influencing factors of NGPCL, scholars commonly utilize social statistical data for analysis. It is generally believed that the quality of resources is a direct influencing factor of NGPCL [35,36], and the production cost triggered by urbanization is the core influencing factor [37,38]. Additionally, farmers’ planting preferences can also affect the occurrence of NGPCL [39,40]. NGPCL also produces various effects: it compresses the space for grain production, which increases the pressure on the regional food supply [41]. Research has also indicated that large-scale NGPCL can have negative impacts on the ecological environment, such as soil hardening and water pollution [42,43]. However, some scholars point out that reasonable NGPCL is a specific practice of sustainable agricultural systems. It can help enhance the region’s water resources’ carrying capacity and ecological benefits [44] and, further, meet the diversified dietary needs of residents [45].

The existing research has provided significant references for the scientific understanding of NGPCL, but there are still some limitations. Firstly, many existing studies rely on the cropland area planted with cash crops as an indirect indicator for the analysis. This research paradigm is one-sided, failing to elucidate the theoretical basis of NGPCL and overlooking its various types. Secondly, land parcels are the fundamental units of cropland utilization, yet most existing studies use administrative regions as research units, relying on social statistical data and lacking related analyses based on a parcel-level perspective. This makes it difficult to reveal the characteristics of NGPCL from a micro perspective. Thirdly, the analysis of relevant influencing factors mostly focuses on socio-economic development and farmer preferences, with fewer scholars analyzing the impact of differences in parcel characteristics on NGPCL from a “cost–revenue” perspective. Fourthly, existing research mainly focuses on economically developed regions, but the risk of NGPCL is relatively higher in mountainous areas due to poorer agricultural production environments [46]. Lechang City is a typical representative of underdeveloped mountainous areas. In recent years, with the continuous advancement of urbanization, a severe loss of agricultural labor has occurred, and the trend of NGPCL in Lechang City has become increasingly apparent. Conducting relevant analysis in Lechang City will help deepen our understanding of the characteristics and driving mechanisms of NGPCL in mountainous areas, providing a case foundation for the formulation of food security policies.

Therefore, this study takes Lechang City as the research area, constructs the definition criteria and measurement index for NGPCL, analyzes the clustering characteristics, typological features, and distribution characteristics of NGPCL at the parcel level, and further explores the driving mechanisms of NGPCL based on the “cost–revenue” perspective. Finally, corresponding policy recommendations are provided based on the research findings. These research results can provide a source of reference for the sustainable utilization of cropland and the control of food security in China and also contribute to a deeper understanding of NGPCL in mountainous areas by global scholars.

## 2. Theoretical Analysis and Research Framework

### 2.1. Theoretical Analysis

**Figure 1:**
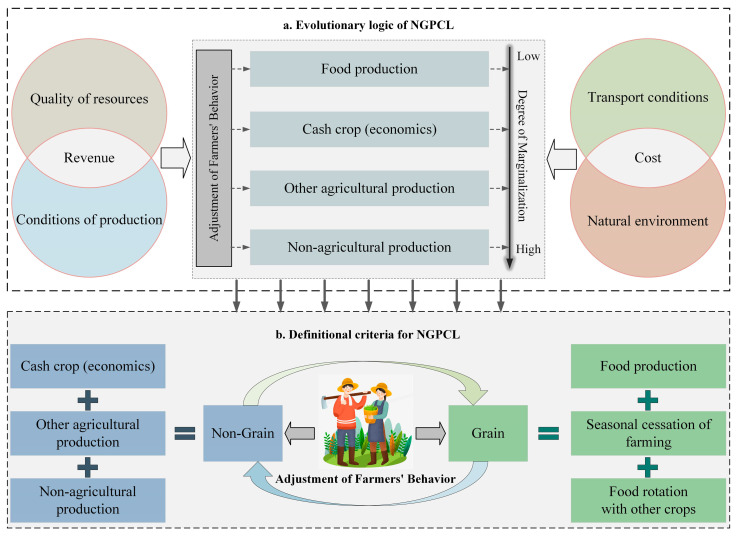
Theoretical basis of NGPCL.

The process of NGPCL can typically be explained by the theory of cropland marginalization. Cropland marginalization refers to the process where the net income from cropland decreases, even to the point where it no longer has beneficial functions and economic value [39,47]. In this process, farmers change their production methods to pursue higher profits [40,48]. The driving mechanism behind NGPCL follows a similar pattern (as shown in Figure 1). Compared to other agricultural production methods, grain production may be less profitable or even unprofitable. Therefore, agricultural producers, in order to attain higher profits, may choose to adjust their cropping structure or change their land use patterns [49].

Among these, the plot serves as the fundamental material unit for cropland utilization. The characteristics of the plot primarily affect farmers’ revenue and costs, thereby influencing the occurrence of NGPCL. The characteristics of the plot mainly include the quality of resources, conditions of production, transport conditions, and the natural environment. The quality of resources determines the yield and quality of crops, while the conditions of production determine the efficiency of agricultural production. These two factors together affect the revenue of cropland utilization. The transport conditions determine the transportation and commuting costs of agricultural production, while the natural environment affects the maintenance costs of cropland utilization. These two factors are important variables influencing the costs of cropland utilization. Therefore, from the perspective of the plot, NGPCL is undertaken by farmers based on the principle of maximizing revenue, considering the costs and benefits influenced by the quality of resources, conditions of production, transport conditions, and the natural environment.

### 2.2. Research Framework

**Figure 2:**
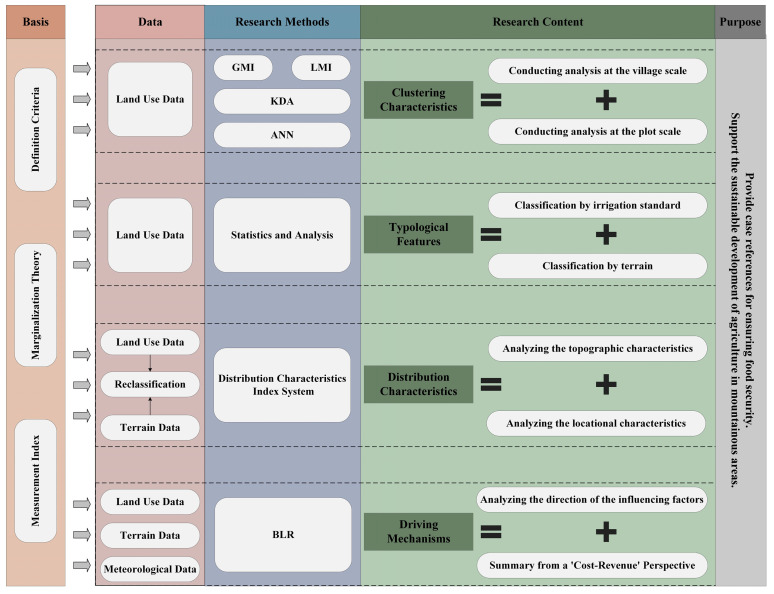
Analysis framework of NGPCL.

This study combines multiple datasets and various analytical models to explore the characteristics and driving mechanisms of NGPCL in Lechang City (as shown in Figure 2). Firstly, based on the theoretical foundation of cropland marginalization, this study constructs the definition criteria and measurement index for NGPCL. Secondly, at both the village and plot levels, this study investigates the clustering characteristics of NGPCL. Additionally, this study explores whether different types of cropland exhibit differences in the extent of NGPCL. Furthermore, by comparing the relationship between the characteristics of the plot and the extent of NGPCL, this study analyzes the distribution characteristics of NGPCL in hilly and mountainous areas. Finally, focusing on the quality of resources, conditions of production, transport conditions, and the natural environment of the plots, this study analyzes the key influencing factors of NGPCL and summarizes its driving mechanisms. Based on the above research findings, this study will formulate corresponding policy recommendations with the aim of providing references and insights for ensuring food security in mountainous areas and the sustainable utilization of cropland.

## 3. Data and Methods

### 3.1. Overview of the Study Area and Data Sources

#### 3.1.1. Overview of the Study Area

**Figure 3:**
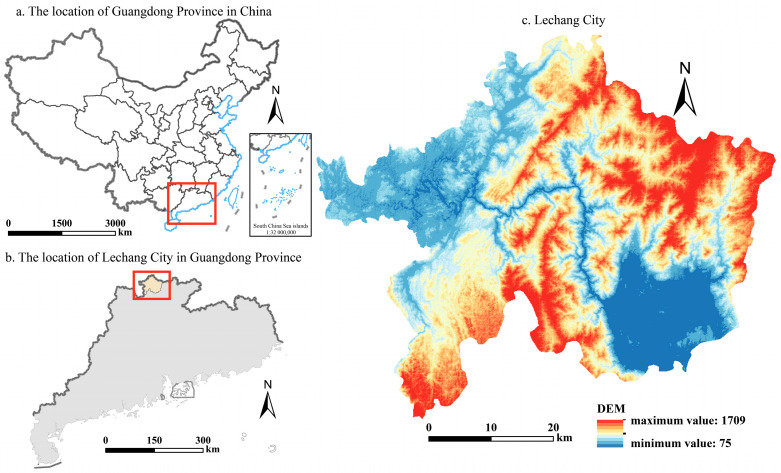
Location of Lechang City.

Lechang City is a county-level administrative region located in the northern part of Shaoguan City, Guangdong Province, China (latitude 24°57′ to 25°31′ N, longitude 112°51′ to 113°34′ E) (as shown in Figure 3), primarily characterized by a subtropical monsoon climate. It is a typical mountainous city, with hills and mountains accounting for 86.5% of its total area. According to the data from the Third National Land Survey, Lechang City has a total of 265.05 km^2^ of cropland. According to official statistics from Shaoguan City in 2022, the regional gross domestic product (GDP) of Lechang City reached USD 3.065 billion in 2021, with the primary industry accounting for 15.93%. This indicates a decrease of 5.04% compared to 2009. The grain output in the county was 89,940.2 tons in 2021, showing a decrease of 29,376 tons compared to 2009, indicating a significant increase in regional food security risks.

#### 3.1.2. Data Sources

The relevant data on land use mainly come from large-scale land survey data and annual updated evaluations of cropland quality. Terrain data are sourced from the ASTER GDEM dataset published by the Geographic Spatial Data Cloud (http://www.gscloud.cn accessed on 1 March 2024), with a data accuracy of 30 m. Meteorological data are obtained from the ERA5 series data released by the European Centre for Medium-Range Weather Forecasts (ECMWF). Detailed data sources are shown in Table 1.

### 3.2. Research Methods

#### 3.2.1. Definition Criteria and Measurement Index

This study combines the diversity and theoretical basis of NGPCL to establish the criteria for defining NGPCL (as shown in Figure 1). Merely discussing NGPCL based on the cultivation of cash crops is one-sided. Focusing solely on changes in cropping structure to some extent overlooks the issue of declining food production in the marginalization process and cannot fully measure the extent of NGPCL [32,50]. Therefore, this study defines all other production activities except for the cultivation of food crops as NGPCL, including the cultivation of cash crops, other agricultural activities, and non-agricultural activities represented by fallowing cropland (as shown in Figure 1b). It is worth noting that crop rotation and seasonal fallowing are resource conservation measures aimed at increasing food production [51]. Therefore, crop rotation and seasonal fallowing should be considered within the scope of food production. Additionally, since this paper focuses on the characteristics of current cropland use, it does not include conversions between cropland and other land types.

Using the “proportion of land area used for grain cultivation in all cropland” as the inverse indicator, we indirectly construct the measurement index of NGPCL to measure the extent of NGPCL in the research unit. The specific calculation method is as follows:

(1) $$\begin{array}{c} N=1-\frac{\left(A+B+C\right)}{D} \end{array}$$

In the formula, *N* represents the measurement index of NGPCL, with higher values indicating a higher degree of NGPCL; *A* represents the area of cropland used for grain cultivation; *B* represents the area of cropland used for crop rotation (being in a state of grain cultivation for at least one quarter of the year); *C* represents the area of cropland left fallow (to maintain cropland productivity, left idle for at least one year or one quarter); *D* represents the total area of cropland within the research unit.

#### 3.2.2. Spatial Autocorrelation

Spatial autocorrelation includes two types: Global Moran’s I (GMI) and Local Moran’s I (LMI). GMI reflects the degree of clustering or dispersion of elements in space. LMI reflects the clustering characteristics of geographic attributes in space, determining the likelihood and location of high-value clustering and low-value clustering [52]. This study uses both GMI and LMI to jointly analyze the clustering characteristics of high and low values of NGPCL at the village scale.

#### 3.2.3. Kernel Density Analysis

Kernel density analysis (KDA) is a non-parametric density calculation method used to estimate the probability density function, which effectively analyzes the degree of local clustering and global clustering characteristics of spatially discrete samples [53]. Given the spatially discrete and numerous but small-sized cropland plots, this study utilizes the ArcGIS 10.2 data platform to convert cropland plot elements in the study area into point features. Then, KDA is applied to investigate the clustering patterns of non-grain production and food-growing at the plot scale (“food-growing” refers to the phenomenon of growing food crops, particularly grains or cereals for human consumption). The specific calculation method is as follows:

(2) $$\begin{array}{c} f\left(x,y\right)=\frac{1}{nh^{2}}\sum_{i=1}^{n}k\left(\frac{d_{i}}{h}\right) \end{array}$$

In the formula, $f\left(x,y\right)$ represents the estimated density of plot aggregation at position (*x*, *y*); *h* is the bandwidth or smoothing parameter; *n* is the number of observations; *k* is the kernel function; $d_{i}$ is the distance from position (*x*, *y*) to the *i*th observed position.

#### 3.2.4. Average Nearest Neighbor Index

The Average Nearest Neighbor (ANN) index is based on the average distance between samples and their nearest neighbors, allowing for comparison between observed and expected values to determine the clustering status of the samples [54]. In this study, this method is applied to investigate the clustering status of different types of cropland under both food-growing and non-grain scenarios. The specific calculation formula is as follows:

(3) $$\begin{array}{c} ANN=\frac{\bar{d_{o}}}{d_{e}}=\frac{\sum_{i=1}^{n}d_{i}/n}{\frac{\sqrt{n/A}}{2}} \end{array}$$

In the formula, $\bar{d_{o}}$ represents the observed average nearest neighbor distance; $d_{e}$ stands for the expected value; $d_{i}$ represents the nearest neighbor distance for the *i*th plot; *n* is the total number of plots; and *A* denotes the total area of the study area in Lechang City. If ANN is less than 1, it indicates a clustered distribution of plots; conversely, if ANN is greater than 1, it suggests a random dispersed distribution of plots.

#### 3.2.5. Classification Criteria

Cropland resources can be classified according to irrigation standards into three categories: dry land, irrigated land, and paddy field. Cropland resources can also be classified based on terrain into three categories: plain, terrace, and slope cropland. By integrating these two classification criteria, a classification system for cropland is established (as shown in Table 2).

#### 3.2.6. Distribution Characteristics Index System

From the plot perspective, NGPCL is inevitably associated with the terrain and location of the plots. Therefore, starting from the dimensions of terrain features and location features, we select six variables to establish the distribution characteristics index system of NGPCL, further elucidating the distribution characteristics of NGPCL (refer to Table 1 for explanations of the variables). Due to variations in statistical units and numerical calibers among the related variables, to eliminate data dimensionality, a reclassification process is employed for the related variable data (as shown in Table 3).

#### 3.2.7. Analysis of Influencing Factors

(1) Selection of influencing factors

**Figure 4:**
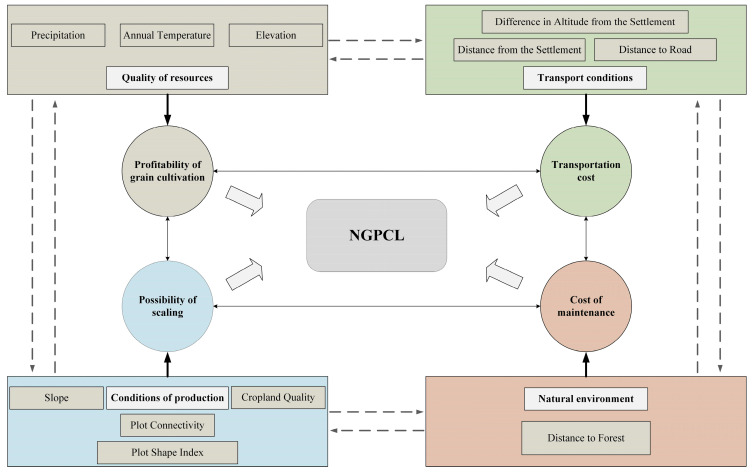
Influencing factors of NGPCL.

Based on the theoretical foundation (as shown in Figure 1) and considering the availability and representativeness of relevant plot characteristics data, this study selected 11 variable indicators to form the influencing factor system for NGPCL (as illustrated in Figure 4).

Indicators related to quality of resources include elevation, annual temperature, and precipitation. These factors determine the thermal environment and irrigation conditions for food crops, directly influencing grain yield and overall revenue from grain production, and thus driving farmers’ behavior towards non-grain production.

Difference in altitude from the settlement, distance from the settlement, and distance to road are indicators related to transport conditions. These indicators affect transportation costs for grain production and subsequently influence farmers’ tendency towards non-grain production.

Indicators related to conditions of production include cropland quality, slope, plot connectivity, and plot shape index. Cropland quality serves as the decisive foundation for grain yield and quality. Slope, plot connectivity, and plot shape index are key influencing factors for farmers to achieve scale grain production. Greater slope, lower plot connectivity, and irregular plot shape make it difficult to establish scale grain production.

Distance to forested areas is a variable related to the natural environment, indicating the degree of wildlife disturbance. The farther the distance to forested areas, the lower the likelihood of wildlife disruption to grain production, resulting in lower maintenance costs for cropland.

(2) Binary logistic regression

Whether plots undergo non-grain production is a binary classification problem. Binary logistic regression (BLR) is widely used in studying farmer behavior and effectively addresses binary decision problems [55]. Therefore, this study chooses BLR to explore the key driving factors of NGPCL.

(4) $$\begin{array}{c} \ln\left(\frac{P_{i}}{{1-P}_{i}}\right)=\alpha +\sum_{j=1}^{m}\beta_{j}x_{ij}+\varepsilon_{j} \end{array}$$

In the formula, $P_{i}$ and $\left({1-P}_{i}\right)$ represent the probabilities of the *i*th plot of cropland belonging to non-grain production and food-growing, respectively; *m* is the total number of influencing factors; $x_{ij}$ denotes the *j*th influencing factor of the *i*th plot of cropland; $\beta_{j}$ is the regression coefficient of the *j*th factor; *α* is the intercept value; and $\varepsilon_{j}$ is the error term.

## 4. Analysis of Results

### 4.1. Clustering Characteristics

#### 4.1.1. Clustering Characteristics from the Perspective of Villages

In Lechang City, a total of 34.92% of cropland is used for non-grain production. Using administrative villages as research units, the area and extent of NGPCL are statistically analyzed, and the natural breaks classification method is adopted to classify the level (as shown in Table 4).

**Figure 5:**
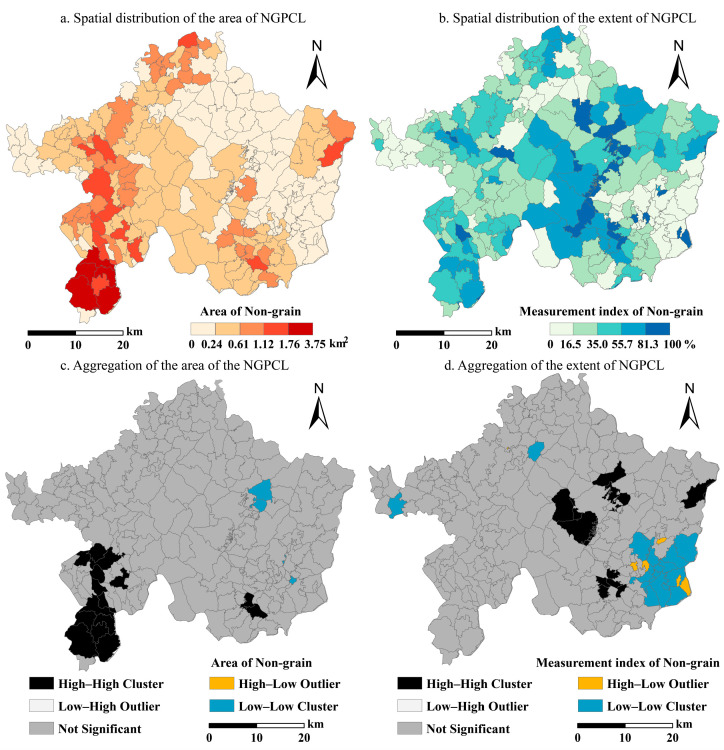
Clustering patterns at the village scale.

Based on the statistical results, we analyzed the spatial pattern of NGPCL. Overall, there is a differentiated distribution pattern between high and low values of the area of non-grain production (as shown in Figure 5a). A total of 52 villages have an area of NGPCL exceeding 0.61 km^2^, mostly located in the southwest to northwest regions of Lechang City. Meanwhile, there are significant differences in the degree of NGPCL among villages (as shown in Figure 5b). A total of 171 villages have a non-grain production measurement index of less than 50%, mainly distributed in the western and eastern parts of the study area.

The results of GMI indicate that both the area and intensity of NGPCL passed the significance test with *p* < 0.01, suggesting that the clustering of these two geographical elements is not occurring by chance. Additionally, the I values for the area and intensity of NGPCL are 0.315 and 0.298, respectively (as shown in Table 5). These results suggest that there is a spatial clustering tendency for both the area and intensity of NGPCL.

Further employing LMI reveals the spatial clustering locations of the area and intensity of NGPCL from the village perspective. Villages with larger areas of NGPCL are forming localized clusters in the southwest of Lechang City. However, most other villages do not exhibit significant clustering patterns in space (as shown in Figure 5c). Regarding the intensity of NGPCL, the central region shows localized “high–high clustering,” while the southeast region primarily exhibits “low–low clustering” as the main clustering characteristic (as shown in Figure 5d).

#### 4.1.2. Clustering Characteristics from a Plot Perspective

**Figure 6:**
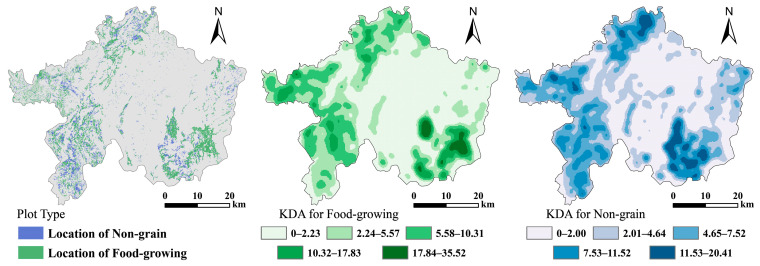
Clustering characteristics at the plot scale.

For food-growing, the KDA index is higher in the southeast and southwest of Lechang City. Regarding non-grain production, the KDA index mainly exhibits a pattern of “high around, low in the middle”. Generally, plots used for food-growing and those for non-grain production show similar clustering patterns, which are also consistent with the distribution pattern of croplands in Lechang City (see Figure 6).

The results of the ANN analysis show that all elements tested passed the significance test with *p*-values less than 0.01, and all ANN values are less than 1. This indicates that all samples are in a clustered state (as shown in Table 6).

Compared to the plots for non-grain production, the plots for food-growing have smaller ANN indices, indicating a stronger tendency towards clustering in space. This is because the labor productivity of food production is positively correlated with operational scale, hence farmers often pursue large-scale operations when engaging in food production [35].

In terms of the plots for non-grain production, terraces exhibit the most significant dispersion. This is because terraces on the periphery are more prone to wildlife disturbances, making them more likely to transition into NGPCL. Regarding plots for food-growing, slope croplands show the most notable dispersion, while plains exhibit the highest degree of clustering. This phenomenon is attributed to the topographical conditions affecting the low-quality slope croplands in mountainous areas, often resulting in higher fragmentation. Conversely, plains resources tend to be more contiguous, displaying a clustered pattern in production and operation.

### 4.2. Typological Features

**Figure 7:**
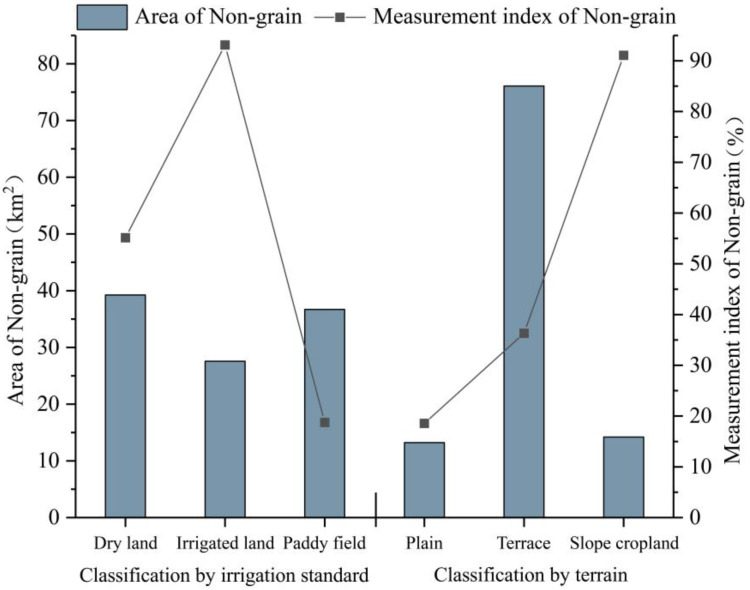
Typological features of NGPCL.

In the overall cropland resources of Lechang City, the proportions of dry land, irrigated land, and paddy fields are 24.02%, 9.99%, and 65.99%, respectively. In terms of the area of NGPCL, dry land exhibits NGPCL on the largest scale, followed by paddy fields, and then irrigated land. Regarding the degree of NGPCL, irrigated land has the highest score, reaching 93.11%. Dry land follows with an index value of 55.13%, and paddy fields have the lowest index value of 18.76% (as shown in Figure 7). The development of this situation may be due to the better irrigation conditions of paddy fields, which can provide a more effective water supply for grain production. Therefore, paddy fields are generally not used for planting cash crops or left fallow. However, dry land and irrigated land are more susceptible to the influence of natural rainfall, resulting in uncontrollable factors in agricultural production and a higher risk of reduced grain yield. Among them, irrigated land is mostly used for vegetable production, hence it exhibits the highest degree of non-grain production among the three types of cropland.

In the overall cropland resources of Lechang City, the proportions of plain, terrace, and slope cropland are 24.03%, 70.72%, and 5.26%, respectively. In terms of area of NGPCL, terraces have NGPCL on the largest scale, while the scale of plains and slope cropland is roughly the same. Regarding the degree of NGPCL, slope cropland has the highest score, approximately 91.10%. Next is terrace, with a score of 36.30%. Lastly, plain has a score of 18.57% (as shown in Figure 7). Due to severe soil erosion and lower cropland quality, slope cropland is categorized as inferior cropland resources. These areas have a higher degree of marginalization and are more prone to non-grain production. Plains and terraces have relatively gentle slopes, which are conducive to large-scale grain production. Plains are particularly suitable for mechanized production and management, making farmers more inclined to engage in grain production on plains.

### 4.3. Distribution Characteristics

#### 4.3.1. Terrain Features

**Figure 8:**
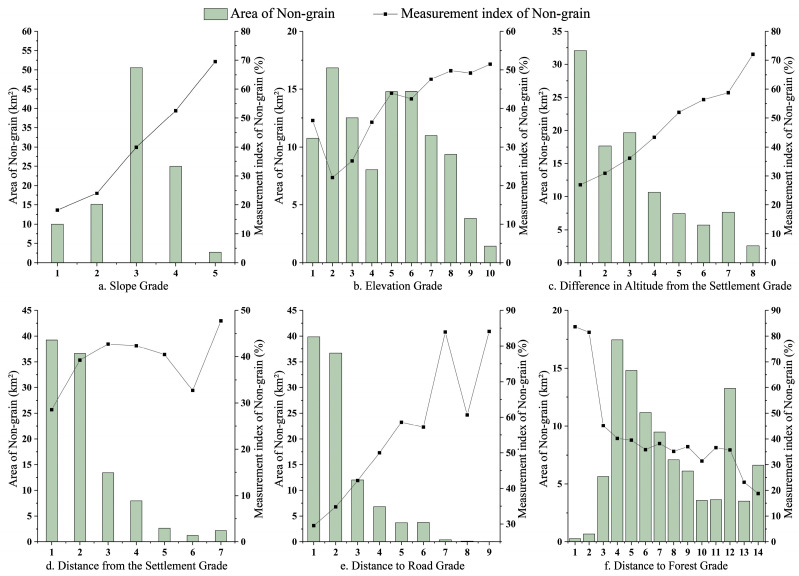
Distribution characteristics of NGPCL.

The degree of NGPCL is significantly positively correlated with the slope. When the slope is greater than 25°, the index of NGPCL reaches as high as 69.52% (as shown in Figure 8a). In practice, cropland with steep slopes is susceptible to the impact of heavy rainfall, leading to surface erosion. Moreover, agricultural machinery is also difficult to operate on cropland with steep slopes. Overall, there is a positive correlation between the degree of NGPCL and the elevation (as shown in Figure 8b). Specifically, when the elevation is less than 200 m, the index of NGPCL decreases rapidly with increasing elevation. When the elevation exceeds 200 m, the index of NGPCL tends to increase with increasing elevation. This phenomenon may be due to restrictions on commuting for farmers, as cropland at higher elevations tends to be abandoned. From a global perspective, the index of NGPCL increases with increasing difference in altitude from the settlement (as shown in Figure 8c).

Overall, the greater the slope, elevation and difference in altitude from the settlement, the more likely NGPCL occurs. This is because under these conditions, farmers are unable to utilize large-scale agricultural machinery for extensive production activities, resulting in relatively lower economic output from the cropland.

#### 4.3.2. Location Features

The index of NGPCL is generally positively correlated with the distance from the settlement (as shown in Figure 8d). This is because an increase in distance from the settlement significantly increases commuting costs. The index of NGPCL shows a fluctuating upward trend with increasing distance to the road (as shown in Figure 8e). Distance to the road is closely related to transportation input factors for grain production. Greater distance implies higher transportation costs, which could affect the profitability of grain production and increase the likelihood of planting other crops. The degree of NGPCL decreases with increasing distance to the forest, with the greatest rate of change occurring in the 0–10 m range (as shown in Figure 8f). This is because forests serve as important habitats for various wildlife species, and cropland closer to forested areas is more susceptible to wildlife disturbances, leading to reduced grain yields.

Combining the above findings, it is evident that there is a clear locational preference for NGPCL. NGPCL is more likely to occur in areas farther from settlements, farther from roads, and closer to forests. Farming in these areas entails higher transportation costs, commuting costs, and maintenance costs. Therefore, the likelihood of NGPCL is higher in these areas.

### 4.4. Driving Mechanisms

To obtain stable analytical results, we first used a linear regression model to diagnose the collinearity among various influencing factors. The results showed that all variables had VIF values less than 10. VIF value is an important indicator to reveal the existence of covariance between multiple variables; when the VIF value of all variables is less than 10, it means that there is no multicollinearity in the combination of variables of linear regression, and the analysis results are reliable. To obtain stable analytical results, we first used a linear regression model to diagnose the collinearity among various influencing factors. The results showed that all variables had VIF values less than 10, indicating a high stability of the variable combinations. The results of BLR indicate that the model has passed the omnibus test for significance, suggesting the reliability of the analysis results (as shown in Table 7).

The *p*-values of annual temperature, distance from the settlement, and distance to forest were all greater than 0.01, indicating no significant impact of these three variables on NGPCL. All other variables passed the significance test with *p*-values less than 0.01, indicating that the remaining influencing factors would have a significant impact on NGPCL (as shown in Table 7). From the B-values, it can be observed that elevation, cropland quality, plot connectivity, and plot shape index would have a negative impact on NGPCL, while difference in altitude from the settlement, distance to road, slope, and precipitation would have a positive impact on NGPCL.

Based on the “cost–revenue” perspective, integrating the analysis results of theoretical foundations and influencing factors, we can summarize the driving mechanism of NGPCL at the plot scale. NGPCL is primarily the production choice of farmers aiming to pursue higher revenue. Among them, the main basis for farmers’ production choices is the balance between cost and revenue. Each influencing factor mainly affects the cost and revenue of grain production, thereby driving farmers to change their production mode and trigger NGPCL.

In general, Lechang City is predominantly mountainous. Typically, higher elevations imply greater solar radiation for crops, which is advantageous for rice to accumulate more flavor substances and sugars. Engaging in grain production at high altitudes is conducive to obtaining higher-quality grain and higher returns. Therefore, elevation has a negative impact on NGPCL. Additionally, high-quality cropland facilitates obtaining high yields of grain, leading to higher profits for farmers. Therefore, cropland quality is also a positive factor influencing NGPCL.

Regularly shaped and contiguous croplands are often more conducive to mechanized production. Farmers using machinery to carry out grain production can effectively increase production efficiency and achieve higher returns per unit of time. Therefore, the plot shape index and plot connectivity of cropland inversely affect NGPCL.

The greater the difference in altitude from the settlement for farming, the higher the commuting costs for farmers. Distance from the settlement is highly positively correlated with transportation costs for farmers. Higher slopes indicate lower possibilities for mechanized production, which increases labor costs. Therefore, difference in altitude from the settlement, distance to road, and slope all affect farmers’ production costs and positively influence NGPCL.

Precipitation affects the maintenance costs of agricultural production. In Lechang City, terraces are the primary form of cropland resources. Heavy rainfall easily causes field collapse, thereby increasing the required input of elements for farmers to maintain cropland.

## 5. Discussions

### 5.1. Research Discussion

This study presents three main innovations. Firstly, it clarifies the evolution logic of NGPCL based on the theory of land marginalization. It further reveals the multi-type nature of NGPCL and introduces its innovative measurement index. Secondly, unlike traditional studies, this research combines land survey data to conduct relevant analyses of NGPCL at the plot level. This approach effectively reveals the characteristics of NGPCL from a micro perspective. The conclusions drawn from such research provide more realistic evidence for the government to effectively manage cropland utilization. Thirdly, previous studies on the influencing factors of NGPCL have mostly focused on socio-economic variables, paying less attention to the impact of plot characteristics on cropland utilization. This study adopts a “cost–revenue” perspective to select relevant influencing variables and combines statistical models to conduct analysis, effectively filling the gaps in related research.

By comparing the conclusions of this study with existing findings, an attempt is made to validate the reliability of the conclusions drawn in this research. This study reveals varying degrees of non-grain production across different types of cropland, which is akin to the characteristics of cropland marginalization [55,56]. Due to the favorable irrigation conditions in paddy fields and the suitability of plains for mechanized production [57,58], paddy fields and plains exhibit lower levels of marginalization and are less prone to NGPCL.

Regarding the distribution characteristics of NGPCL, Chen found a clear terrain preference for NGPCL in basic farmland [59]. Additionally, similar distribution characteristics of cropland abandonment in mountainous areas were also observed [60]. The conclusions of these scholars are consistent with the findings of this study, indicating that the extent of NGPCL varies with changes in terrain and location conditions. This study also found that NGPCL exhibits significant clustering characteristics, with terraces having a lower degree of clustering compared to slope cropland. This phenomenon may firstly be attributed to the large-scale abandonment of poor-quality slope cropland in mountainous areas [61]. Secondly, because marginal terraces have lower economic benefits and higher production costs, they are more prone to NGPCL [62].

In terms of the factors influencing NGPCL, due to differences in regional climate conditions and farming practices, the elevation in the study area has a negative impact on NGPCL. This conclusion differs from the marginalization mechanism of cropland in the southwestern mountainous areas [63], indicating the complexity and regional differences in the formation mechanism of NGPCL. Therefore, the management and governance of NGPCL should be tailored to local conditions, and corresponding strategies should be implemented accordingly.

At the same time, it is worth noting that the availability and quality of data often determine the reliability of research conclusions. The land survey data used in the study are obtained through a nationwide unified survey by national-level agencies, ensuring high reliability. The remaining data also come from official scientific research institutions, effectively guaranteeing data quality. The above data are also commonly used in related studies [14,64]. Therefore, high-quality research data can also confirm the reliability of the conclusions of this study.

The article clarifies the theoretical basis of NGPCL, innovates the measurement index for NGPCL, and reveals the multi-type characteristics and driving mechanisms of NGPCL. However, this study focuses on the influence of plot characteristics on farmers’ operational choices and lacks relevant discussion on policy factors. In fact, national policy constraints and administrative incentives have significant impacts on agricultural production decisions. Future research will pay more attention to the relationship between regional policies and NGPCL, while establishing studies on NGPCL from different scale perspectives, and attempt to analyze the characteristics and formation mechanisms of NGPCL from multiple angles.

### 5.2. Recommendations for Countermeasures

In response to the findings of this study, this research proposes the following policy recommendations with the aim of providing guidance for the management of NGPCL and the sustainable utilization of cropland in mountainous areas.

(1) Optimize cropland irrigation facilities to enhance the quality of cropland

Research indicates that different types of cropland exhibit varying levels of non-grain production. Therefore, efforts should be made to optimize water storage facilities and improve the layout of ditches and canals. Simultaneously, irrigation facilities should be further improved to mitigate the impact of natural precipitation on grain production. There should also be a strong push for the transformation of slope cropland to prevent the concentration of large-scale abandonment of low-quality slope cropland. Additionally, attention should be paid to the effects of natural factors such as heavy rainfall. It is essential to actively optimize the maintenance and supervision mechanisms of terraces to prevent field collapse caused by heavy rainfall and subsequent loss of grain yield. In summary, efforts should be focused on enhancing the overall quality of cropland resources and increasing farmers’ income from grain production.

(2) Promote mechanized production suitable for the local conditions to enhance the efficiency of grain cultivation

Research shows that topographical and locational conditions are key factors constraining the input of mechanical elements. Therefore, on one hand, measures such as leveling field surfaces and consolidating field plots should be taken to optimize the mechanized environment of cropland. On the other hand, it is necessary to further improve transportation networks and widen roads for machinery access to mountainous areas. This can effectively reduce commuting and transportation costs for farmers. Additionally, conventional large-scale machinery cannot be used in mountainous and hilly regions. Therefore, there should be a strong push to introduce multifunctional small-scale agricultural equipment. Environmental improvement and the introduction of suitable machinery can effectively address the marginalization of grain production in mountainous areas, thereby increasing farmers’ income from grain production.

(3) Improving the agricultural production structure promotes the sustainable development of cropland in mountainous areas.

Reasonable NGPCL not only enhances the stability of agricultural systems but also increases farmers’ income. Therefore, further improvements should be made to the agricultural production structure. Firstly, with the aim of ensuring food security, grain production areas should be designated based on the actual situation of cropland resources in each region, ensuring sufficient grain output. Secondly, local governments should aim to revitalize cropland resources and allow unused cropland in other areas to be utilized for alternative agricultural activities. Measures such as agricultural subsidies should be implemented to encourage farmers to develop idle cropland resources. For instance, in mountainous areas where there is a large-scale abandonment of inferior slope cropland, the government could permit farmers to plant economic trees on these slopes and provide technical or financial support. This approach not only prevents the idle use of cropland resources but also increases farmers’ income sources. However, it is worth noting that all agricultural production and development must follow the premise of protecting cropland resources. The local government should actively fulfil its supervisory role and reasonably restrain the extent and intensity of cropland development by farmers.

## 6. Conclusions

This study focuses on Lechang City as the research area and utilizes various geographic and spatial methods and econometric models to comprehensively reveal the clustering, typological, and distributional characteristics of NGPCL. This study also identifies key influencing factors and summarizes the driving mechanisms behind NGPCL. The specific conclusions are as follows:

(1) In Lechang City, 34.92% of cropland across the entire area is used for non-grain production. At the village level, villages with larger areas of NGPCL are locally clustered in the southwest of Lechang City, while villages with higher degrees of NGPCL are mainly clustered in the central part of Lechang City. At the plot level, compared to the plots for non-grain production, the plots for food-growing tend to be more clustered spatially. Regarding the plots for non-grain production, terraces exhibit the most significant dispersion pattern, followed by slope cropland, and finally plains. For the plots for food-growing, slope cropland shows the most significant dispersion pattern, while plains exhibit the highest degree of clustering.
(2) In terms of irrigation types, the largest scale of NGPCL occurs in dry land, followed by paddy fields, and, finally, irrigated land. However, the highest degree of NGPCL is observed in irrigated land, followed by dry land, and then paddy fields. Regarding terrain classification, terraces have the largest scale of NGPCL, while the scales of plains and slope cropland are roughly similar. The highest degree of NGPCL is observed in slope cropland, followed by terraces, and finally plains.
(3) NGPCL exhibits clear preferences for topography and location. The greater the slope, elevation, and difference in altitude from the settlement, the more likely NGPCL occurs. Additionally, NGPCL is more likely to occur in areas farther from settlements, farther from roads, and closer to forests.
(4) Apart from the three variables of annual temperature, distance from settlement, and distance to forest, all other variables significantly influence NGPCL. Elevation, cropland quality, plot connectivity, and plot shape index all affect the profitability of grain production, serving as negative drivers for NGPCL. Difference in altitude from the settlement, distance to road, slope, and precipitation all impact the cost of grain production, exerting positive effects on NGPCL. NGPCL is primarily the production choice of farmers aiming to pursue higher profits. Various influencing factors primarily affect the costs and revenues of grain production, thereby driving farmers to change their production patterns and initiate NGPCL.

## Figures and Tables

**Table 1:** Content and sources of data.

Data Source	Data Name	Variable Content
Land Survey Data	Cropland Plot Data	0 = Planted with crops, 1 = Cropland used for non-grain production
	Difference in Altitude from the Settlement	Difference in altitude between the plot and the nearest residential area. Measured in meters
	Distance from the Settlement	Distance from the plot to the nearest residential area. Measured in meters
	Distance to Road	Distance from the plot to the nearest road. Measured in meters
	Plot Connectivity	Area size of the individual plot. Measured in square meters
	Plot Shape Index	Geometric shape of the plot with reference to a square. Calculated as 0.25 × perimeter/area (power, 0.5)
	Distance to Forest	Distance from the plot to the nearest forest patch. Measured in meters
ASTER GDEM	Elevation	Average elevation of cropland measured per plot. Measured in meters
	Slope	Slope of the cropland plot
ERA5	Annual Temperature	Sum of daily average temperatures accumulated per plot over one year. Measured in degrees Celsius
	Precipitation	Total daily precipitation sum, averaged over one year. Measured in millimeters
Cropland Quality Annual Update	Cropland Quality	Indicator based on organic matter content. Measured in grams

**Table 2:** Classification system of cropland.

Classification Criteria	Types of Croplands	Content
Classification by irrigation standard	Dry land	Cropland that relies solely on natural precipitation to provide water for crops
	Irrigated land	Cropland near natural reservoirs but highly susceptible to the impact of natural rainfall
	Paddy field	Cropland with artificial irrigation water supply
Classification by terrain	Slope cropland	Cropland distributed on slopes with poor flatness and severe soil erosion
	Terrace	Terraced fields constructed along contour lines on hillsides, featuring strip-shaped terrace or undulating cross-sectional profiles.
	Plain	Flat farmland with a relatively level terrain.

**Table 3:** Distribution characteristics index system.

Category	Variable Name	Data Processing
Terrain Features	Slope	Slope < 2° assigned 1, 2–6° assigned 2, 6–15° assigned 3, 15–25° assigned 4, >25° assigned 5
	Elevation	Every 100 m is one level, with 10 levels above 900 m
	Difference in Altitude from the Settlement	Levels 1–2, every 5 m within; levels 3–6, every 10 m within; Level 7: (50–100 m); Level 8: (100 m or more)
Location Features	Distance from the Settlement	Within levels 1–3, every 300 m is one level; within levels 4–6, every 600 m is one level; Level 7 is 2700 m or more
	Distance to Road	Within levels 1–5, every 100 m is one level; within levels 6–8, every 500 m is one level; Level 9 is 2000 m or more
	Distance to Forest	Level 1 distance is 0 m; within levels 2–11, every 10 m is one level; within levels 12–14, every 50 m is one level; Level 14 is 200 m or more

**Table 4:** Criteria for classifying NGPCL.

Types/Hierarchy	Extremely Low	Low	Medium	High	Extremely High
Area (km^2^)	(0, 0.24]	(0.24, 0.61]	(0.61, 1.12]	(1.12, 1.76]	(1.76, 3.75]
Proportions (%)	(0, 16.5]	(16.5, 35.0]	(35.0, 55.7]	(55.7, 81.3]	(81.3, 100]

**Table 5:** Test results of Global Moran’s I at the village scale.

Typology	I-Value	*p*-Value	Z-Value	Variance
Area of NGPCL	0.315	0.00 *	9.743	0.001
Measurement index of NGPCL	0.298	0.00 *	8.998	0.001

0.00 * Indicates that the value is less than 0.01 (consistent with the following).

**Table 6:** The results of ANN.

Production Status	Type of Cropland	Expected Distance	Observed Distance	ANN Index	Z-Value	*p*-Value
Plots for non-grain production	All plots	168.566	314.391	0.536	−88.374	0.00 *
	Plain	266.397	802.350	0.332	−47.968	0.00 *
	Terrace	208.404	389.218	0.535	−71.492	0.00 *
	Slope cropland	356.379	674.680	0.528	−40.755	0.00 *
Plots for food-growing	All plots	168.681	322.870	0.522	−87.620	0.00 *
	Plain	177.699	544.644	0.326	−63.288	0.00 *
	Terrace	199.179	383.105	0.520	−74.236	0.00 *
	Slope cropland	1174.452	1823.145	0.644	−10.848	0.00 *

**Table 7:** The results of BLR.

Omnibus: *p* = 0.00 *						
Variable Type	Variable Name	B-Value	Standard Error	*p*-Value	VIF	EXP(B)
Quality of resources	Elevation	−0.119	0.018	0.00 *	7.821	0.888
	Annual Temperature	0.000	0.000	0.770	6.327	1.000
	Precipitation	0.003	0.000	0.00 *	1.550	1.003
Transport conditions	Difference in Altitude from the Settlement	0.068	0.010	0.00 *	1.640	1.070
	Distance from the Settlement	0.004	0.011	0.736	1.343	1.004
	Distance to Road	0.065	0.014	0.00 *	1.368	1.067
Conditions of production	Cropland Quality	−0.106	0.012	0.00 *	2.075	0.900
	Slope	0.329	0.022	0.00 *	2.390	1.389
	Plot Connectivity	−13.827	0.832	0.00 *	1.316	0.000
	Plot Shape Index	−0.217	0.033	0.00 *	1.269	0.805
Natural environment	Distance to Forest	0.012	0.005	0.034	1.900	1.012
Constant		−3.470	0.619	0.00 *	—	0.031

## Data Availability

The data in this study are available from the corresponding authors upon request. Due to the sensitivity of the study area, some data cannot be made public.
